# Bridging science and curriculum: preparing future leaders in computational toxicology

**DOI:** 10.3389/ftox.2025.1662963

**Published:** 2026-01-12

**Authors:** Frances Hall, Candice Johnson

**Affiliations:** Instem, Stone, United Kingdom

**Keywords:** compound safety, computational toxicology, qualitative structure-activity relationships (QSAR), read-across, toxicology education

## Abstract

Computational toxicology plays an important role in chemical safety assessments. Computational methods are applied to early-stage screening in drug discovery, hazard identification, and regulatory safety assessment. This article presents an overview of the foundational skills, technical capabilities and regulatory literacy recommended to successfully apply and evaluate (Q)SAR ((Quantitative) Structure-Activity Relationship) methodologies (e.g., statistical and alert-based approaches) and read-across within established frameworks such as the (Q)SAR Assessment Framework (QAF), OECD validation principles and context-specific regulatory frameworks; for example, ICH M7. Additionally, the manuscript covers strategies that can be used to integrate theoretical and practical experience with foundational skills (e.g., internships, case studies, regulatory simulations). An overall educational framework that emphasises competency-based education through interdisciplinary exposure is presented. The framework outlines the progression from foundational knowledge to methodological understanding, context of use application and the ability to assess the reliability of outcomes. Although the integrated framework is applicable to both regulatory and non-regulatory use contexts, the manuscript presents regulatory focused use cases, which could be explored within educational settings. These use cases consider mature, as well as emerging regulatory applications, and therefore highlight the need to apply foundational principles (e.g., expert review, qualification of methods) in diverse contexts. This approach reinforces a context-of-use driven approach to curriculum design and provides opportunities for growth through real-world application and experiential learning, supported by collaborative initiatives and open-access resources.

## 1 Introduction

Computational toxicology is an essential field within the life sciences. It provides support for the evaluation of chemical toxicity across multiple sectors (such as industrial chemicals, pharmaceuticals, food and consumer products). Computational toxicology encompasses a range of modelling approaches including *in silico* models integrated with biological data to assess toxicological effects. *In silico* predictions are derived from chemical structure representations and (quantitative) structure–activity relationship ((Q)SAR) approaches, which provide a link between chemical structure and biological activity. The use of (Q)SAR increases the efficiency of toxicity assessments and contributes to reducing reliance on animal testing.

Computational toxicology plays a critical role in accelerating chemical or drug discovery and development. It is a non-testing approach which offers significant advantages by reducing the costs associated with a compound’s synthesis and experimental testing. These methods are applied in evolving regulatory frameworks, such as the EU Registration, Evaluation, Authorisation and Restriction of Chemicals (REACH) (European Chemicals Agency ECHA, 2025) and the FDA’s Predictive Toxicology Roadmap (US Food and Drug Administration FDA, 2025b), which emphasize the use of alternative methods to animal testing. The field leverages machine learning, cheminformatics, and systems biology, resulting in several distinct advantages, including early anticipation of toxicological outcomes in the R&D pipeline, efficient prioritization of compounds for testing, and fulfilling regulatory requirements.

This publication explores the specific skills, knowledge, and educational strategies that are needed for building capacity in computational toxicology, with emphasis on regulatory applications. This manuscript is intended to support a broad range of educational experiences such as, educators developing coursework in computational toxicology, regulatory agencies building *in silico* training programs, and academic institutions designing degree pathways, as well as students entering the field. The manuscript describes core concepts, methodologies, as well as practical use case applications. The aim is to provide a practical basis for curriculum development and professional training, which is aligned with current and emerging demands in regulatory science and toxicological risk assessment.

This manuscript is novel in that it integrates the context-of-use (primarily regulatory) directly into an educational framework, offering an approach not commonly addressed in existing literature. The framework illustrates how understanding and application interact across methods, context-of-use, and educational strategy, providing a cohesive structure for teaching and learning. It also reinforces the value of industry–academia collaboration, incorporating an industry voice to ground educational practices in real-world needs. By centering the context-of-use, the framework naturally accommodates the expansion into AI methodologies, ensuring relevance as technologies evolve. Furthermore, the comparative method presented in Table 1 serves as a practical resource for establishing foundational knowledge.

**TABLE 1:** High-level comparison between statistical-based methods, expert rules-based methods and read-across methods in computational toxicology.

Feature	Statistical-based methods	Expert rules-based methods	Read-across
Approach	Models that link chemical descriptors to biological/toxicological outcomes	Rules based on mechanisms and/or empirical observations to infer a biological/toxicological outcome	Data extrapolation based on similarity across multiple similarity domains (e.g., structure, ADME, reactivity, toxicological)
Output	Quantitative (including potency categories and probability scores), which may be interpreted qualitatively using thresholds (e.g., toxic vs. non-toxic)	Qualitative or categorical predictions; including potency categories (e.g., toxic/non-toxic, strong skin sensitizer/non skin sensitizer, CPCA categories for N-nitrosamines)	Qualitative or quantitative with expert-supported justification (e.g., hazard class, point of departure estimates)
Explainability	High when the results can be explained using the model metadata such as model features, training examples, analogues that are made accessible and interpretable; limited for *post hoc* explanations	High; rules are explicit, usually mechanistically informed and transparent	Interpretability and explainability must be strong to support the acceptability of the read-across outcome. Similarity should be clearly explained, justified, and interpreted across different domains to ensure the outcome is reliable and associated with limited uncertainty
Data requirement	Requires well-curated training data with high-quality experimental outcomes; the quantity and type of data depend on endpoint complexity, chemical diversity, and intended application	Requires a curated knowledge base and well-defined structural alerts linked to known mechanisms of toxicity; rules or alerts can also be identified using chemoinformatics approaches that detect specific chemical patterns associated with a particular toxicity, even without in-depth knowledge of the underlying mechanism. In general, predictivity may be limited where underlying mechanisms are poorly understood or data are sparse	Requires high-quality data for structurally and mechanistically similar source chemicals, and additional supporting data to assess the similarity across the different domains (e.g., ADME). Statistical- and expert-rules based predictions may be integrated to support the read-across justification. Chemoinformatics methods may support structural similarity evaluation, including analogue identification
Regulatory use	Yes – accepted in screening and regulatory frameworks (e.g., ICH M7) with reference to the OECD validation principles for regulatory acceptance. Expert review is a critical element in achieving regulatory acceptance	Yes – widely used for regulatory hazard identification and screening (e.g., ICH M7) with reference to the OECD validation principles for regulatory acceptance. Expert review is a critical element in achieving regulatory acceptance	Acceptance depends heavily on depth of scientific justification. Moreover, read-across involves some subjective interpretation of similarity, which represents a significant hurdle limiting its regulatory acceptance. Additionally, acceptance also depends on the specific endpoint being assessed and the context of use
Strengths	• Assesses all areas of the molecule, including whole molecular properties • Can often explain the structural basis for the prediction • Quick to generate a model • Fast to compute a prediction	• Capable of precisely identifying reactive groups or toxicophores that are linked to a mechanism • Includes reasons for reactivity (e.g., bioactivation, activation and deactivation) • Fast to compute a prediction • Better at predicting positive outcomes	• Has potential applicability in predicting complex *in vivo* outcomes where there is data on suitable analogs
Limitations	• No direct mechanism provided, but may be elucidated as part of the (Q)SAR explanation • Difficult to model complex *in vivo* outcomes • Limited applicability to newer chemical domains (e.g., drug impurities with structures similar to those of newly developed APIs)	• Usually localized to the presence of a substructure and often does not take into account the whole molecule • Difficult to make negative predictions • May miss novel mechanisms • Difficult to model complex *in vivo* outcomes	• Depends heavily on analogue quality and justification • Manual methodology that relies heavily on subjective interpretation, making results difficult to reproduce • Time consuming to generate and fully document the results

### 1.1 Applied use cases in regulatory and product development contexts

The use of computational toxicology is increasingly embedded across regulatory submissions, product development processes, and hazard classification frameworks. The following practical use cases illustrate how *in silico* methods are applied across various sectors and toxicological endpoints.

#### 1.1.1 Product quality and safety

Various computational platforms, both freeware and commercial, are available to support toxicological assessments. For example, commercial platforms such as Leadscope, Derek Nexus and Case Ultra, and open-source tools such as OECD QSAR toolbox, VEGA and OPERA, may facilitate regulatory submissions by providing access to curated databases and supporting predictions for bacterial mutagenicity and carcinogenicity of pharmaceutical impurities, extractables and leachables (E&L), animal health products, and plant protection agents. This is aligned with regulatory frameworks such as:

- ICH M7: (International Council on Harmonisation–Multidisciplinary 7) Recommends complementary (statistical and expert rule-based) (Q)SAR models for mutagenic impurity risk assessment in human pharmaceuticals (ICH, 2017).
- Carcinogenic Potency Calculation Approach: The US FDA, EMA, and Health Canada have recently issued guidance related to handling *N*-nitrosamine impurities and degradants, including the Carcinogenic Potency Category Approach, which is a decision tree to derive Acceptable Intake (AI) limits for N-nitrosamines using defined structure activity relationship rules that account for both activating and mitigating effects (US Food and Drug Administration FDA, 2023).
- Use of International Standard ISO 10993-1 to support applications to FDA: Encourages the use of structure-activity modeling to better understand the carcinogenicity potential of contact materials in medical devices. This assessment should consider both mutagenic and non-mutagenic modes of actions (US Food and Drug Administration and Use of International Standard ISO 10993-1, 2023).
- Veterinary Medicinal Products: Adopts (Q)SAR-based principles similar to ICH M7 for mutagenic impurity evaluation in animal health (European Medicines Agency, 2020).
- ICH Q3E (unpublished at the time of publication): Expected to define procedures supporting the toxicological evaluation of E&L chemicals (ICH, 2020).

#### 1.1.2 Early discovery and candidate screening

*In silico* models play a key role in deprioritizing toxic candidates early in the R&D pipeline, potentially reducing the risk of late-stage failures. Toxicophore identification is used to refine chemical structures. Furthermore, early screening assessments support preclinical testing strategy development, improves decision-making and enables resource allocation.

#### 1.1.3 Non-genotoxic impurities

The ICH Q3A and Q3B guidelines address the qualification of non-genotoxic impurities. The use of computational tools and read-across approaches for the assessment of non-mutagenic impurities is of high interest; for example, the draft EMA reflection paper (European Medicines Agency, 2024) recommends the use of (Q)SAR and read-across approaches to assess impurities that are above qualification thresholds.

#### 1.1.4 Abuse liability

Substances with central nervous system (CNS) activity often require an assessment for abuse potential. *In silico* profiling of abuse liability and blood brain barrier permeability can facilitate earlier screening in the discovery phase, complement traditional testing methods, and support regulatory decisions (Faramarzi et al., 2022; U.S. FDA, 2023).

#### 1.1.5 Classification, labelling and packaging

*In silico* toxicology predictions offer an efficient approach to address data gaps (where biological information is unavailable) in toxicity and safety information required for the classification and labelling of chemicals, including those transported or registered under regulatory frameworks. The time and cost involved make it challenging to generate toxicity data using traditional methods. *In silico* predictions support compliance with regulatory programs such as the EU REACH regulation (European Chemicals Agency ECHA, 2025) and the US Toxic Substances Control Act (TSCA) (U.S. EPA, 2025), which increasingly recognize predictive models and expert review as integral components of hazard evaluation. The endpoints which are assessed may include mutagenicity, carcinogenicity, skin and respiratory sensitization, irritation (skin, eye, and respiratory), reproductive and developmental toxicity, acute toxicity, endocrine disruption and repeated dose toxicity.

#### 1.1.6 Occupational risk assessment

Occupational risk assessment evaluates worker safety by assessing chemical hazard. Computational toxicology supports this process through predictive models, read-across and exposure estimation when experimental data are limited or lacking. Such information could be used to assign occupational exposure bands (OEB) and communicate potential health hazards which determine appropriate handling; for example, selection of appropriate personal protective equipment (PPE) (Massarsky et al., 2024; Graham et al., 2022).

#### 1.1.7 Drug–drug interactions (DDI)

The 2020 FDA guidance on drug-drug interactions provides criteria for determining when *in vitro* studies are needed to assess the inhibitory effects of a metabolite on cytochrome P450 (CYP) enzymes, including potential for mechanism-based inhibition (MBI) (US Food and Drug Administration, 2025a). Structural alerts for mechanism-based inhibition, as well as (Q)SAR models for both reversible and irreversible CYP inhibition could be used to define workflows which support the guideline (Faramarzi et al., 2025).

#### 1.1.8 Food flavourings and pesticide residues

*In silico* methods, such as (Q)SAR and read-across, are used to predict the genotoxicity of flavourings and pesticide residues, as highlighted by European Food Safety Authority (EFSA) publications (Younes et al., 2021; EFSA, 2016a). These approaches are also used for prioritizing and conducting preliminary toxicological assessments in the risk evaluation of food contact materials, particularly concerning impurities and non-intentionally added substances (NIAS) like reaction and degradation products (EFSA, 2016b). Additionally, the US FDA’s Center for Food Safety and Applied Nutrition (CFSAN) incorporates structural similarity considerations for toxicity prediction in the safety assessment of food contact substances and their components (US FDA CFSAN, 2025).

#### 1.1.9 Weight-of-evidence (WoE) assessments

Computational toxicology plays an important role in assembling WoE assessments (Bassan et al., 2024) for complex endpoints such as carcinogenicity and reproductive and developmental toxicity. Integrating *in silico* predictions with experimental evidence can increase the robustness of an assessment (Myatt et al., 2018).

The use cases presented here focus primarily on pharmaceutical and chemical regulatory applications. However, computational toxicology is more widely applied across additional sectors including cosmetics, agrochemicals, and industrial chemicals. Within these sectors, there are evolving regulatory contexts which could be supported by computational methods.

## 2 Understanding and teaching computational toxicology

Computational toxicology generally includes (Q)SARs and other modelling approaches such as Physiologically Based Kinetic (PBK) modelling. The technical concepts discussed here are specific to (Q)SAR approaches, which involve the use of computer-based models; that is, an *in silico* system, and data analysis techniques to predict the toxic effects of chemical substances on living organisms, based on information derived from their chemical structure. The following discussion presents foundational concepts.

### 2.1 Chemical structures and descriptors

A computational toxicology assessment uses a 2-D representation of a chemical structure, typically encoded as a SMILES (Simplified Molecular Input Line Entry System) string or drawn within a structural file (e.g., SDF (Structure Data File) or MOL (part of the MDL-file format family)). From this input, a variety of descriptors (quantitative representations of molecular properties) are calculated. These include both physicochemical descriptors, such as molecular weight, logP (octanol-water partition coefficient), and polar surface area, as well as structural descriptors that capture the presence or absence of specific substructures (Myatt et al., 2018). Chemical fingerprints are binary or hashed representations of molecular features and are commonly used to encode the presence or absence of specific substructures or patterns (Yang et al., 2022). Fingerprints (e.g., MACCS (Molecular ACCess System) keys, ECFP (Extended-Connectivity Fingerprint), or proprietary fingerprint sets transform chemical input data into standardized formats and enable similarity-based methods and machine learning models (Myatt et al., 2018). In addition, more complex features (topological, electronic, quantum mechanical) may be generated to encode structural information for use in machine learning or statistical models. These descriptors form the foundation for predictive modeling approaches such as (Q)SARs, which link specific combinations of molecular features to biological or toxicological outcomes.

### 2.2 Methodologies in *in silico* toxicology

#### 2.2.1 Statistical-based models ((Q)SAR)

(Q)SAR models are mathematical models that relate chemical structure to biological activity. Statistical models are trained on curated datasets of chemical structures with known biological activity or toxicological outcomes, which are called training sets. (Q)SAR models use descriptors; such as, physicochemical properties, or structural features to describe the relationship between chemical structure and activity (Myatt et al., 2014). A model is built using one or more molecular descriptors from every chemical to predict the toxic response. A trained (Q)SAR model is then used to predict the activity of untested compounds based on their structural characteristics. Common statistical modeling approaches include Partial Logistic Regression, Partial Least Squares Regression, Random Forests, Neural Networks (including deep learning variants), Support Vector Machines (SVM) and k-Nearest Neighbors (k-NN) (Bassan et al., 2024). Method selection is often based on data characteristics, need for interpretability, and use case (including scalability based on computational resources) (Myatt et al., 2014).

#### 2.2.2 Expert rule-based models

Expert rule-based models operate using curated sets of conditional logic rules (e.g., “if an aromatic nitro group is present, then potential mutagenicity is flagged”) (Myatt et al., 2014; Dearden et al., 1997). These models are built upon mechanistic toxicology principles which are encoded as structural alerts, which are chemical substructures associated with specific toxicological outcomes. Development of rule-based systems typically involves assembling and curating toxicological reference datasets, qualifying and refining alerts, and identifying new alerts through the examination of new or proprietary data (Myatt et al., 2017).

Expert rule-based systems are transparent, interpretable, and add mechanism-based reasoning. However, they may be limited when the underlying mechanism is unknown, as with complex toxicological endpoints, or the chemical space is poorly represented in the knowledgebase. They can be difficult to interpret in the context of negative results, as the absence of an alert may reflect a lack of mechanistic knowledge rather than a prediction of no effect.

Adherence to OECD principles (OECD, 2007) including a defined endpoint, an unambiguous algorithm, a defined domain of applicability, and appropriate measures of goodness-of-fit and predictivity is critical for regulatory acceptance of both statistical-based models and expert rule-based systems.

#### 2.2.3 Read-across approaches

Read-across is an expert driven evaluation of toxicity that can leverage statistical or expert rules based approaches, but remains a distinct methodology (and is thus a separate column in Table 1). Read-across is a non-testing method used to predict the toxicity of a target chemical by identifying and extrapolating data from structurally and mechanistically related chemicals (source analogs) with known toxicity (Dimitrov and Mekenyan, 2010; van Leeuwen et al., 2009; Cronin et al., 2011; Cronin et al., 2013).

Key aspects of read-across include identifying relevant data-rich analogs and evaluating similarity with the intent of deriving a robust hypothesis that supports the read-across outcome. The read-across outcome is supported by evidence that the target chemical and selected analogs either share common toxicological pathways or lack activity. Computational models can be used to support analog identification and the assessment of similarity profiles across multiple domains; such as structural, physicochemical, metabolic, and toxicological domains. When combined with QSAR, expert rules-based models, and other relevant NAMs (New Approach Methodologies), read-across assessments are strengthened for use in regulatory decision-making (Rovida et al., 2025; Rovida et al., 2021).

### 2.3 Expert review

Expert review is an important aspect of *in silico* toxicology, particularly in regulatory contexts. Computational models provide predictions based on chemical structure and training/reference data; however, an evaluation of model predictions is prudent to ensure and communicate prediction reliability (Myatt et al., 2017).

For a comprehensive discussion on assessing and improving prediction reliability through expert review, the reader is referred to Myatt et al. (2018). For brevity, the following are items considered as part of an expert review.

- Evaluating model outputs, including confidence scores, consensus between different methodologies, and resolving any potential conflicting predictions
- Reviewing underlying training data, structural analogs, features flagged as important by the model and model applicability domains
- Assessing mechanistic plausibility by considering known toxicological pathways
- Integrating multiple lines of evidence which may include *in silico*, *in vitro*, and *in vivo* data where available

An expert review considers the limitations of each methodology, including the reliability of training data, out-of-domain predictions, and metabolic activation/deactivation. While the expert review is inherently subjective, it is recognized as an important step for deriving reliable and scientifically defensible predictions. The expert review is especially important in regulatory submissions, where any uncertainty must be transparently documented and communicated (Myatt et al., 2018; Amberg et al., 2019; Barber et al., 2015).

### 2.4 Building competency in computational toxicology

**FIGURE 1:**
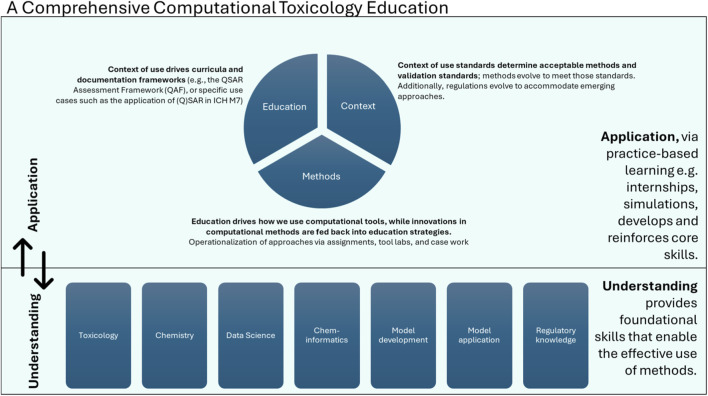
A comprehensive computational toxicology education facilitates interpretation and domain fluency, which consequently enables good application of the theory. The educational strategy, which is based upon real-world applications, develops and reinforces the skills required for understanding.

Developing competency in computational toxicology requires interdisciplinary training. It is important for users of computational tools to have a comprehensive understanding of modeling techniques and toxicological principles. This involves a combination of skills which includes the ability to critically evaluate the underlying toxicity data and understand how they influence the model output. Knowledge of the (regulatory) context of application is also critical, as it defines the optimal way to integrate *in silico* predictions into chemical safety assessments. Building this skill set is essential for evaluating the reliability of a prediction. Figure 1 presents a conceptual framework that outlines the progression from foundational understanding to practical application in computational toxicology education. It highlights the interdisciplinary skills that are required and also illustrates how these competencies are operationalized through education. A comprehensive computational toxicology education facilitates interpretation and domain fluency, which consequently enables good application of the theory.

Each *in silico* methodology has strengths and limitations and these are profiled in Table 1. Rather than focusing solely on how individual tools function, educational programs should also emphasize the methodological approaches, including statistical-based methods, expert rule-based systems, and read-across (Table 1). A good understanding of the strengths and weaknesses of *in silico* methodologies is important as it enables users to select appropriate methods, evaluate model outputs, and understand when a prediction is of sufficient reliability. In addition to enabling the interpretation of model results, this knowledge also facilitates efficient communication with interdisciplinary teams that may include toxicologists, chemists, modelers, and regulatory scientists.

The demand for non-animal toxicity assessments continues to grow (National Institutes of Health, 2025). Therefore, there is a need to cultivate a workforce which is fluent in the principles and practices of NAMs, including, computational toxicology. This includes an understanding of machine learning algorithms, chemical descriptor generation and interpretation, structural alert interpretation, and the integration of *in silico* results into weight-of-evidence assessments. Additionally, as regulatory agencies continue to evaluate and adopt the use of computational methods in safety assessment frameworks, it is important that scientists are prepared to meet these evolving standards through both theoretical understanding and application.

## 3 Practical implementation of computational toxicology

### 3.1 Industry expectations and desired skills for computational toxicologists

Computational toxicology requires knowledge of toxicological principles and also the ability to interpret computational results, particularly when integrating diverse data types (Myatt et al., 2018). An understanding of key toxicological endpoints; for example, mutagenicity, carcinogenicity, reproductive and developmental toxicity, acute toxicity, sensitization as well as ADME (absorption, distribution, metabolism, and excretion) enables professionals to interpret the biological relevance of computational outputs (Myatt et al., 2018). It is therefore beneficial to educate from both theoretical and practical points of view. As such, educators can embed case studies (Johnson et al., 2022), mechanistic frameworks such as adverse outcome pathways (AOPs (Leist et al., 2017)), and toxicological principles into coursework.

As data availability increases, data science capabilities are increasingly valuable. In addition to the interpretation of prediction results, being able to manage, analyze, and visualize complex datasets, and apply machine learning or artificial intelligence (AI) based methods to extract meaningful patterns and/or predictions is advantageous. Exercises that include the analysis of datasets can provide exposure to data harmonization procedures. Data harmonization processes are used to standardize data across various sources and study types, where applicable. Experience developing a well curated dataset could reinforce the practical importance of this process for extracting reliable results. Collaboration between departments, such as informatics and toxicology departments, can help bridge disciplinary gaps and provide students with technical experience.

As computational toxicology is often applied within regulatory contexts, an understanding of which computational approaches could be used to support chemical registration and safety decisions is beneficial. As demonstrated in Figure 1, knowledge of regulatory or context of use frameworks determines the selection of a model, documentation requirements, and expert interpretation. Simulating regulatory submissions for mock assessments and including a computational toxicology component into regulatory science modules can provide practical exposure. Training in science communication also forms an important component in explaining expert review and read-across outcomes. That is, the ability to clearly explain model predictions, and associated uncertainties and limitations, is an important skill. A solid foundation in chemistry, including familiarity with physicochemical and structural descriptors (as outlined in Section 3.1), supports meaningful interpretation of model outputs. There are several opportunities to build these competencies, including, internships, interdisciplinary team projects, presentations, and peer review exercises. As programs are more aligned with technical, regulatory, and collaborative requirements students can be expected to contribute meaningfully to the next-generation of predictive toxicology.

### 3.2 Collaborative approaches to education and training

Market leaders in computational toxicology include private and public companies, public consortia, government and academic institutions. Some companies have supported academic training through initiatives such as academic licensing, providing training resources, and partnerships that support course development. Public initiatives and regulatory bodies have developed open-access tools and datasets that serve as valuable resources for educational needs. Platforms such as the National Toxicology Program’s Integrated Chemical Environment tools and data sets (ice.ntp.niehs.nih.gov), and the OECD toolbox (https://www.oecd.org/en/data/tools/oecd-qsar-toolbox.html) offer access to data and various methodologies. These platforms provide students and professionals with practical exposure to computational tools. Cross-industry collaborations and professional societies can also create opportunities to gain practical experience with the computational methodologies. Consortia and collaborative initiatives, such as EU-ToxRisk (https://cordis.europa.eu/project/id/681002/reporting), ONTOX (https://ontox-project.eu/), and HESI (https://hesiglobal.org/about-hesi/) demonstrate how research initiatives support early-career scientists through fellowships, postdoctoral training, sharing technologies, and institutional expertise. Guest lectures by experienced professionals as well as professional workshops can also provide exposure to fundamental skills in regulatory or use case applications. In these contexts, Figure 1 provides a framework for designing training pipelines that connect foundational understanding with applied experience.

Notably, academic and research institutions contribute to the advancement of the field through the study of adverse outcome pathways (AOPs), development of new methodologies and approaches, and more recently, the use of AI into predictive frameworks (Hartung and Kleinstreuer, 2025; Hartung and Tsaioun, 2024; Scheufen et al., 2025; Watanabe-Sailor et al., 2019; Sinitsyn et al., 2022; Arnesdotter et al., 2021; Belfield et al., 2025; Roberts and Aptula, 2014).

A variety of collaborative strategies can help reinforce collaboration and ensure sustainable training pathways. These strategies include training pipelines that link academic learning with applied experience, as well as fellowship, mentorship and internship opportunities which connect to real-world experiences. Additionally cross-sector dialogue to keep educational content aligned with evolving regulatory and industry needs is valuable. Such opportunities provide students and young professionals with the experience needed to meet current and future needs in computational toxicology.

### 3.3 Regulatory considerations

It is important for practitioners to understand how computational toxicology methods are adopted into regulations. Tools with regulatory acceptance must be built on curated, high-quality data, demonstrate reliability, and be linked to a clearly defined toxicological endpoint. In line with established best practices and validation principles, such as those outlined by the OECD in the (Q)SAR Assessment Framework (OECD, 2023) criteria such as the model’s domain of applicability, algorithm, and validation must be documented. It is also important to note that computational models are updated as new data or knowledge becomes available and, as such, models may be refined over time (Hasselgren et al., 2020). Knowledge of the full lifecycle of computational methods (including development to use case application, e.g., regulatory use) prepares individuals to be agile participants in an evolving regulatory and application landscape. As illustrated in Figure 1, use case knowledge is a critical component of computational toxicology education that can be emphasized curriculum design.

### 3.4 Emerging use of artificial intelligence (AI) tools

The future of computational toxicology is influenced by regulatory evolution, and technological advancement. Traditional AI and machine learning approaches have long supported predictive toxicology; however, the integration of more broadly defined AI models into predictive frameworks requires careful implementation (Hartung and Kleinstreuer, 2025). Regulatory preference is for models that support transparency (a clearly defined endpoint, interpretability, validation) and overall regulatory readiness (FDA. U.S. Department of Health and Human Services; EMA/CHMP/CVMP/83833/2023, 2024). In January 2025, the FDA issued draft guidance on the use of AI to support regulatory decision making. The guidance outlines a risk-based credibility framework for evaluating AI models that considers the context of use, an assessment of model risk, and documentation of metadata to establish the AI model credibility and adequacy within the use context (FDA. U.S. Department of Health and Human Services, 2025). There is growing interest and potential for the application of AI in drug discovery, development and toxicological research (Hartung and Kleinstreuer, 2025; National Center for Toxicological Research NCTR, 2024; Sinha et al., 2023) although careful integration with scientific and regulatory principles will be required to successfully integrate broadly defined AI approaches into regulatory decision-making frameworks (Hartung and Kleinstreuer, 2025). Nonetheless, the educational framework presented, which includes an analysis of application standards based on context of use (e.g., regulatory and discovery applications), theoretical understanding and practical experience is applicable towards educational objectives pertaining to the responsible use of AI. Given the increasing regulatory and industry focus on AI model transparency and credibility, integrating these principles into computational toxicology education is essential to prepare trainees to responsibly develop, evaluate, and apply predictive models in both research and regulatory contexts. For additional examples of the use of AI in toxicology and risk assessment see Haßmann et al. (2024), Bueso-Bordils et al. (2024) and Luechtefeld and Hartung (2025).

## 4 Conclusion

To fully realize and propel advancements in computational toxicology (including (Q)SAR, expert rule-based models, read-across methodologies as well as emerging AI-driven approaches), educational frameworks must evolve in parallel. In addition to aptitude with existing and emerging tools, being able to evaluate prediction reliability, interpret results and communicate model outputs are important. This skill set requires interdisciplinary exposure that includes lessons and experiences relevant to toxicology, chemistry, data science, and regulations. The educational framework presented in Figure 1 supports the development of interdisciplinary competencies necessary for advancing computational toxicology.

Imparting students and early-career scientists with a strong conceptual foundation and exposure to real-world use cases is valuable. The field of computational toxicology depends on collaboration between academia, industry, and regulators. Similarly, the effort to cultivate a workforce that can advance computational toxicology responsibly and effectively requires interdisciplinary education and practical training.
